# 

**Published:** 1936

**Authors:** 


### The Medical Library of the University of Bristol,

WITH WHICH IS INCORPORATED

**The Library of the Bristol Medico-Chirurgical Society.**

*The following donations have been received since the publication of the list in October*, 1936.

January, 1937.

| | | |
|---|---|---|
| Professor R. J. Brocklehurst (1) | .. .. | 1 volume. |
| Carnegie Institution, Washington (2) | .. .. | 1 ,, |
| Professor E. W. Hey Groves (3) | .. .. | 9 volumes. |
| Dr. F. S. Hunter (4) | .. .. .. .. | 1 volume. |
| H. K. Lewis & Co., Ltd., Publishers (5) | .. | 1 ,, |
| Professor C. Bruce Perry (6) | .. .. .. | 1 ,, |
| Royal College of Surgeons of England (7) | .. | 1 ,, |
| Michell Clarke Fund (8) | .. .. .. .. | 1 ,, |

Unbound periodicals have also been received from Professor E. W. Hey Groves, Professor C. Bruce Perry and Dr. J. Odery Symes.

### THE ONE HUNDRED AND FIFTY-SEVENTH LIST OF BOOKS.

The figures in round brackets refer to the figures after the names of the donors. The books to which no such figures have been attached have either been bought from the Library Fund or received through the *Journal*.

| | | | |
|---|---|---|---|
| **Abbott, M. E.** .. .. | *Atlas of Congenital Cardiac Disease* .. .. | | 1936 |
| **Aykroyd, W. R.** .. | *Vitamins and other Dietary Essentials* | 2nd Ed. | 1936 |
| **Bainbridge and Menzies.** | *Essentials of Physiology.* Ed. Hartridge | 7th Ed. (1) | 1931 |
| **Bainbridge and Menzies.** | *Essentials of Physiology.* Ed. Hartridge | 8th Ed. | 1936 |

| | | | |
|---|---|---|---|
| **Benedict, F. G.** .. | *The Physiology of the Elephant* .. .. | (2) | 1936 |
| **Bertwistle, A. P.** .. | *A Descriptive Atlas of Radiographs* 3rd Ed. | (3) | 1936 |
| *British Pharmacopœia,* 1932: Addendum, 1936 .. .. .. .. | | (3) | 1936 |
| **Coleman, F.** .. .. | *Extraction of Teeth* .. .. .. 3rd Ed. | (5) | 1933 |
| **Coppleson, V. M., and D. Miller.** | *Clinical Handbook* .. .. | (3) | 1936 |
| **Goidsenhoven, F. van, and Hoet, J. P.** | *Lessen over de onderzoeksmethoden bij inwendige Ziekten* .. .. .. .. .. | (4) | 1936 |
| **Havens, Leon C.** .. | *The Bacteriology of Typhoid, Salmonella and Dysentery Infections and Carrier States.* Ed. Maxey .. .. .. .. .. .. .. | (8) | 1935 |
| **Jameson, W. W., and Parkinson, G. S.** | *Synopsis of Hygiene* 5th Ed. | | 1936 |
| **Kienböck, R.**.. .. | *Röntgendiagnostik der Knochen und Gelenkkrankheiten,* Heft 4 .. .. .. | (3) | 1936 |
| **Lange, M.** .. .. | *Die Wirbelgelenke* .. .. .. 2te Aufl. | (3) | 1936 |
| **Power, d'Arcy, ed.** | *British Masters of Medicine* .. .. .. .. | | 1936 |
| **Reichel, P.** .. .. | *Die Nachbehandlung nach Operationen* 3te Aufl. | (3) | 1936 |
| **Saegesser, M.** .. | *Chirurgische Operationslehre* .. .. .. | (3) | 1935 |
| **Snowman, J.** .. | *Manual of Emergencies* .. .. 3rd Ed. | (3) | 1936 |
| **Verth, M. zur** .. | *Behandlung der Verletzungen und Eiterungen an Finger und Hand* .. .. 2te Aufl. | (3) | 1936 |
| **Williamson, Bruce** | *A Handbook on Diseases of Children* 2nd Ed. | (3) | 1936 |
| **Wright, Samson** .. | *Applied Physiology* .. .. .. .. 6th Ed. | | 1936 |

TRANSACTIONS, REPORTS, JOURNALS, ETC.

Medical Directory for 1937 .. .. .. .. .. .. .. .. .. .. ..

St. Bartholomew's Hospital, Reports, Vol. LXIX .. .. .. ..

Smithsonian Institution, Washington, Annual Report for 1935 ..

Royal College of Surgeons of England, Calendar for 1936 .. (7)

### Publications Received

From MESSRS. EDWARD ARNOLD & CO.:

*A System of Clinical Medicine.* By T. D. SAVILL, M.D. 10th Edition. Edited by AGNES SAVILL, M.D., and E. C. WARNER, F.R.C.P. Price 28s.

*An Introduction to General Practice.* By E. KAYE LE FLEMING, M.A., M.D. Price 5s.

From MESSRS. BAILLIÈRE, TINDALL & COX:

*A Theory of Cancer and Mnemotherapy.* By RUDOLF ROOSEN, M.D. Price 3s. 6d.

*Aids to Diagnosis and Treatment of Diseases of Children.* By F. M. B. ALLEN, M.D., M.R.C.P. Price 4s. 6d.

From MESSRS. JOHN BALE, SONS & DANIELSSON LTD.:

*Diagnosis of some Delusional Insanity Types in General Practice.* By EDWIN HOPEWELL-ASH, M.D. Price 2s. 6d.

From MESSRS. H. K. LEWIS & Co. LTD.:

*Arthritis in Women.* By R. FORTESCUE FOX, M.D., F.R.C.P., F.R.Met.Soc. Price 2s. 6d.

*Elementary Pathology.* By KEITH S. THOMPSON, M.R.C.S. Price 10s. 6d.

*The Prevention and Treatment of Disease.* By W. M. STEVENS, M.D. Price 2s.

*Historical Notes on Psychiatry.* By J. R. WHITWELL, M.B. Price 10s. 6d.

From MESSRS. E. & S. LIVINGSTONE, EDINBURGH:

*Text-Book of Medicine.* Edited by J. J. CONYBEARE, M.C., M.D., F.R.C.P. Third Edition. Price 21s.

*Illustrations of Regional Anatomy,* VI, VII. By E. B. JAMIESON, M.D., Price 7s. 6d. and 10s.

From OXFORD UNIVERSITY PRESS:

*Operative Surgery.* By ALEX. MILES, M.D., LL.D., F.R.C.S., and D. P. D. WILKIE, M.D., F.R.C.S. 2nd Edition. Price 21s.

*Pathological Physiology and Clinical Description of the Anemias.* By W. B. CASTLE, M.D., S.M., and G. R. MINOT, M.D., S.D., F.R.C.P. Edited by H. A. CHRISTIAN, A.M., M.D., LL.D., Sc.D. Price 15s.

*Graduated Muscular Contractions.* By Sir MORTON SMART, M.D.

From MESSRS. JOHN WRIGHT & SONS:

*British Journal of Surgery.* Vol. XXIV. No. 94. Price 12s. 6d.

### Local Medical Notes

EXAMINATION RESULTS.

**University of Bristol.**—Students of the University have recently passed the following examinations:—

M.D.—*Pass:* H. E. Pearse.

M.B., CH.B.—*Second Examination.* (*Section I*) *Pass:* G. S. Andrews, J. L. Ashley, Marie L. Bartlett, R. C. Blackney, J. Brodie, Joyce W. Brown, N. J. Brown, J. D. Cardale, Jyotikana Datta, J. H. P. Davies-Sage, Cecil M. Drillien, B. L. Finzel, S. J. V. Gilson, Hester M. Hammond, Kathleen M. A. Harland, Dorothy Hughes, W. G. H. Hunt, Mary Jacob, D. J. Jones, Mary E. Loewe, D. S. Maunsell, Phyllis D. Moses, H. Mullen, A. H. M. Oakes, Christine M. Rendell, F. B. Richards, A. F. Rogers, A. R. Rowe, M. O. Skelton, J. C. Valentine, G. A. Walton, J. H. Wilkins.

M.B., CH.B.—*Final Examination.* (*Section I.*) *Pass:* E. M. Barber, P. A. Bennett, K. J. V. Carlson, Joan E. Mackworth, P. C. C. Phelps.

M.B., CH.B.—*Final Examination.* (*Section II.*) *Pass:* Coralie W. Rendle-Short (with Distinction in Obstetrics and Public Health), L. Schnipelsky, E. Want, Ursula G. Hewitt, P. Zimmering.

B.D.S.—*Second Examination.* *Pass:* A. A. Grant, J. R. D. Gordon.

B.D.S.—*Third Examination.* *Pass:* F. P. Ewens (with Distinction), D. R. Stevens, R. J. Gething.

B.D.S.—*Final Examination.* *Pass:* C. A. Blanden, C. C. B. Plumley, P. R. Quartley.

L.D.S.—*Second Examination.* *Pass:* F. W. Clift, E. L. Manton, G. H. Hudson.

L.D.S.—*Third Examination.* *Pass:* F. S. S. Baguley, C. F. Howell, J. B. Inverdale, C. C. Moore, A. J. H. Orchard, J. C. F. Rolls, J. G. Windmill, R. V. Woods, P. H. S. Paine, D. M. Sanders, J. F. Durie, Peggy Rooke.

L.D.S.—*Final Examination.* *Pass:* M. J. Banbury.

**Conjoint Board.** — M.R.C.S., L.R.C.P. — *Pass: In Pathology:* Barbara Cunningham, R. L. J. Derham, M. E. M. Herford, F. J. W. Hooper, C. R. G. Howard. *In Surgery:* J. R. G. Damrel, E. J. Lace. *In Midwifery:* P. W. M. Martin, Mabel W. N. Tribe. *In Anatomy:* Megan E. Jones. *In Physiology:* Joan S. Miller, J. H. G. Morris, Megan E. Jones, C. J. R. Jacob. *In Pharmacology and Materia Medica:* Eileen R. Atchison.

L.D.S., R.C.S.—*Final Examination.* *Pass:* *Part I.:* C. A. Blanden.

APPOINTMENTS.

**Bristol Royal Infirmary.**—Maurice E. Disney, M.D., M.R.C.P., Medical Registrar. F. Wilfred Willway, M.D., M.S., B.Sc., F.R.C.S., Surgical Registrar.

Dr. Elliott T. Glenny, M.B., B.S., has been appointed Medical Instructor, Air Raids Precaution Department, Home Office, for South Wales Area, Cardiff Centre, and Mr. E. M. Pearse, M.R.C.S., F.R.C.P., to a similar post at Salisbury.

---

As we go to press we regret to hear of the death of Mr. A. Walbank, M.B., Ch.B., F.R.C.S., D.O.M.S., Honorary Surgeon to the Bristol Eye Hospital.

### Index

Abdominal pain in childhood.—W. Sheldon, 219.
Acetyl-choline in Treatment of Tobacco Amblyopia.—B. H. Cragg, 237.
Appointments :—
J. T. Chesterman.—Travelling Fellowship in Medical Science, Medical Research Council, 122.
K. H. Pridie.—Assistant Surgeon to Orthopædic Department, Bristol Royal Infirmary, 122.
M. E. Disney.—Medical Registrar, Bristol Royal Infirmary, 122.
F. W. Willway.—Surgical Registrar, Bristol Royal Infirmary, 122.
R. T. Cooke.—Assistant Clinical Pathologist, Department of Preventive Medicine, University of Bristol, 185.
C. A. H. Gee.—Clinical Lecturer in Diseases of Children, University of Bristol, 185.
L. C. Oliver.—Recognized Teacher in Surgery, University of Bristol, 185.
P. Phillips.—Recognized Teacher in Obstetrics, University of Bristol, 185.
C. A. Ashford. — Lecturer in Physiology, University of Bristol, 185.
Miss J. Wiseman.—Assistant Bacteriologist, Department of Preventive Medicine, University of Bristol, 185.
H. Tout.—Colston Research Fellow in Social Survey, University of Bristol, 185.
W. Melville Capper.—Senior Resident Medical Officer, Bristol Royal Infirmary, 185.
L. C. Oliver. — Resident Surgical Officer and Registrar, Bristol General Hospital, 185.
E. T. Glenny.—Medical Instructor, Air Raids Precaution Department, 265.

Blood-serum, ultra-microscopic examination of.—B. A. Peters, 17.
Bristol Eye Hqspital, 35
Bristol Medical Dramatic Club.—A. L. Flemming, 239.
Bristol Royal Infirmary, new Sisters' Hostel, 176.
Bristol University Expedition to Central Asia, 257.
British National Hereditary Council, 117.
Bronchus, carcinoma of the :—
Clinical features.—C. Bruce Perry, 125.
Radiologically considered. — G. B. Bush, 131.
Bronchoscopic diagnosis.—G. Scarff, 137.
Pathological aspect.—A. L. Taylor, 139.
Treatent of.—D. Wood, 145.
Bulleid, A.—Somerset Lake Villages (Long Fox Memorial Lecture), 187.
Bush, G. B.—Carcinoma of the bronchus, Radiologically considered, 131.

Carcinoma of the Bronchus, 125.
Colston Medical Research Fellowships, 57.
Corneal Transplantation, its history, development, and practice.—J. W. Tudor Thomas, 75.
Cragg, B. H.—Treatment of tobacco-amblyopia by acetyl-choline, 237.

Dental Hospital, New Buildings, 259.
Dramatic Club, Bristol Medical, history of.—A. L. Flemming, 239.

Faculty of Medicine, New Buildings and Dental Hospital, 259.
Field, C. E., and Rogers, H.—A fatal case of influenzal meningitis, 151.
Flemming, A. L.—History of the Bristol Medical Dramatic Club, 239.

Graded milk, 54.

Health Services, National: Position of Voluntary Hospitals. — R. F. Barclay, 39.
Health Services, Scottish, Report of Committee on, 163.

Hospitals :—

Bristol Eye, 35.

New Dental, 259.

Winford Orthopædic, 179.

Voluntary, 39.

Iles, A. E.—Photography and Medicine (Presidential Address), 3.

Joseph Rogers Prize, 177.

King George V, In Memoriam, 1.
King George VI, Accession of, 257.

Library Notes, 62, 119, 181, 263.
Local Medical Notes, 66, 122, 185, 265.
Long Fox Memorial Lecture.—A. Bulleid, on Somerset Lake Villages, 187.

Medicine, Faculty of, New Buildings, 259.
Meningitis, a fatal case of influenzal.—C. E. Field and H. Rogers, 151.
Milk, graded, 54.
Minister of Health, visit to Bristol, 178.
Morgan, Conwy Lloyd.—Obituary, 59.

Obituary :—
Morgan, Conwy Lloyd, 59.

Perry, C. Bruce.—Carcinoma of the bronchus, Clinical features, 125.
Peters, B. A.—Blood-serum, ultra-microscopic examination of, 17.
Photography and Medicine.—A. E. Iles (Presidential Address), 3.
Presidential Address. — A. E. Iles, Photography and Medicine, 3.

Queen Victoria Jubilee Convalescent Home, new President of, 54.

Reviews of books, 45, 101, 168, 247.
Rogers, H.—See Field, C. E. and Rogers, H.
Royal Medical Benevolent Fund Centenary, 175.

Scandinavia, surgical clinics in.—A. R. Short, 157.
Scarff, G.—Carcinoma of the bronchus, bronchoscopic diagnosis, 137.
Schrötter, Leopold Von, Centenary of Birth of, 260.
Sciatica, a treatment for.—A. R. Short, 87.
Scottish Health Services, report of the committee on, 163.
Sheldon, W.—Abdominal pain in childhood, 219.
Short, A. R.—A treatment for sciatica, 87.
Short, A. R. — Surgical Clinics in Scandinavia, 157.
Social Survey of Bristol, 258.
Societies :—
Bristol Medico-Chirurgical, 61, 118, 180, 260.
Galenicals, 66, 180.
Somerset Lake Villages.—A Bulleid (Long Fox Memorial Lecture), 187.
Sphenoidal sinus infection, fatal case of influenzal meningitis due to.—C. E. Field and H. Rogers, 151.

Taylor, A. L. — Carcinoma of the bronchus, Pathological aspect, 139.
Thomas, J. W. Tudor.—Corneal transplantation, its history, development, and practice, 75.
Tobacco amblyopia, Treatment of by acetyl-choline, B. H. Cragg, 237.

Voluntary Hospitals, position in the National Health Services, 39.

Williams, Joseph, 58, 117.

Winford Orthopædic Hospital, extensions at, 179.

Wood, D.—Carcinoma of the bronchus, Treatment of, 145.

